# Fructo-oligosaccharides and intestinal barrier function in a methionine–choline-deficient mouse model of nonalcoholic steatohepatitis

**DOI:** 10.1371/journal.pone.0175406

**Published:** 2017-06-20

**Authors:** Kotaro Matsumoto, Mayuko Ichimura, Koichi Tsuneyama, Yuki Moritoki, Hiromichi Tsunashima, Katsuhisa Omagari, Masumi Hara, Ichiro Yasuda, Hiroshi Miyakawa, Kentaro Kikuchi

**Affiliations:** 1 Department of Gastroenterology, Teikyo University Mizonokuchi Hospital, Takatsu-ku, Kawasaki-city, Kanagawa, Japan; 2 Department of Food Science and Nutrition, Nara Women's University, Kita-Uoya Nishimachi, Nara-city, Nara, Japan; 3 Department of Pathology and Laboratory Medicine, Institute of Biomedical Sciences, Tokushima University Graduate School, Tokushima-city, Tokushima, Japan; 4 Department of General Medical Practice and Laboratory Diagnostic Medicine, Akita University Graduate School of Medicine, Akita-city, Akita, Japan; 5 Department of Nutrition, Faculty of Nursing and Nutrition, University of Nagasaki, Nagayo-cho, Nishi-Sonogi-gun, Nagasaki, Japan; 6 The Fourth Department of Internal Medicine, Teikyo University Mizonokuchi Hospital, Takatsu-ku, Kawasaki-city, Kanagawa, Japan; Medizinische Fakultat der RWTH Aachen, GERMANY

## Abstract

Impairments in intestinal barrier function, epithelial mucins, and tight junction proteins have been reported to be associated with nonalcoholic steatohepatitis. Prebiotic fructo-oligosaccharides restore balance in the gastrointestinal microbiome. This study was conducted to determine the effects of dietary fructo-oligosaccharides on intestinal barrier function and steatohepatitis in methionine–choline-deficient mice. Three groups of 12-week-old male C57BL/6J mice were studied for 3 weeks; specifically, mice were fed a methionine–choline-deficient diet, a methionine–choline-deficient diet plus 5% fructo-oligosaccharides in water, or a normal control diet. Fecal bacteria, short-chain fatty acids, and immunoglobulin A (IgA) levels were investigated. Histological and immunohistochemical examinations were performed using mice livers for CD14 and Toll-like receptor-4 (TLR4) expression and intestinal tissue samples for IgA and zonula occludens-1 expression in epithelial tight junctions. The methionine–choline-deficient mice administered 5% fructo-oligosaccharides maintained a normal gastrointestinal microbiome, whereas methionine–choline-deficient mice without prebiotic supplementation displayed increases in *Clostridium* cluster XI and subcluster XIVa populations and a reduction in *Lactobacillales* spp. counts. Methionine–choline-deficient mice given 5% fructo-oligosaccharides exhibited significantly decreased hepatic steatosis (p = 0.003), decreased liver inflammation (p = 0.005), a decreased proportion of CD14-positive Kupffer cells (p = 0.01), decreased expression of TLR4 (p = 0.04), and increases in fecal short-chain fatty acid and IgA concentrations (p < 0.04) compared with the findings in methionine–choline-deficient mice that were not administered this prebiotic. This study illustrated that in the methionine–choline-deficient mouse model, dietary fructo-oligosaccharides can restore normal gastrointestinal microflora and normal intestinal epithelial barrier function, and decrease steatohepatitis. The findings support the role of prebiotics, such as fructo-oligosaccharides, in maintaining a normal gastrointestinal microbiome; they also support the need for further studies on preventing or treating nonalcoholic steatohepatitis using dietary fructo-oligosaccharides.

## Introduction

Nonalcoholic fatty liver disease (NAFLD) is an organ phenotype in metabolic syndrome that is often complicated by obesity, dyslipidemia, and diabetes mellitus [1]. Nonalcoholic steatohepatitis (NASH) is part of the spectrum of NAFLD, combined with hepatic inflammation and fibrosis, leading to cirrhosis [2]. It was reported that the 5-year cumulative incidence of liver cancer in Japanese patients with NASH and cirrhosis was 20% [3]. Regardless of the examination of many researchers, it is not apparent which factor promotes the progression of NAFLD to NASH, and, therefore, a radical curative method is unknown.

According to an analysis of the microbial flora and a study of metabolic products, intestinal bacteria participate in organic homeostasis and pathological changes in the living body [4]. Abnormality of the microbial flora is called dysbiosis, which denotes a lack of diversity of intestinal bacteria because of a disorder of quantitative and qualitative balance [5]. Additionally, in NASH, dysbiosis has been determined via fecal examination, and it is caused by an unbalanced diet or obesity, suggesting that improvements of the microbial flora are correlated with improvements of hepatic steatosis [6].

Dysbiosis leads to biological and immunological intestinal barrier dysfunction through shortages of nutrients in intestinal epithelial cells and mucosal immune deficiency [7–9]. Recently, intestinal barrier dysfunction was noticed in the pathogenesis of NAFLD [10]. A key component of the onset of NASH is the influx of a large amount of pathogen-associated molecular patterns (PAMPs) through disrupted intestinal mucosal epithelium [11, 12] and Kupffer cell hypersensitivity to PAMPs in the liver [13].

After some animal experiments and clinical trials, it was revealed that amelioration of dysbiosis improves intestinal barrier function, indicating that this strategy could represent an effective treatment for certain diseases [14]. Prebiotics are food components that are not digested or absorbed in the upper gastrointestinal tract. They are fermented selectively by beneficial types of intestinal bacteria, favorably altering the composition of microbial flora and conferring healthy effects on both the gastrointestinal tract and entire body of the host [15]. Prebiotics such as oligosaccharides and dietary fibers have such effects, and, in particular, fructo-oligosaccharides (FOSs) meet all of the requirements to serve as probiotics. In this study, to examine whether FOSs can be used to treat NASH, we hypothesized that FOSs can improve dysbiosis and delay the onset of NASH, and examined the hypothesis using a NASH animal model.

## Materials and methods

### Generation of the NASH mouse model

Six methionine–choline-deficient diet (MCD) mice (12-week-old male C57BL/6J mice, Sankyo Labo Service Co. Inc., Tokyo, Japan) were fed an MCD (A02082002B, Research Diets Inc., New Brunswick, NJ, USA) and purified water for 3 weeks and housed under conventional conditions. In addition, six FOS-treated MCD (MCD + FOS) mice were additionally administered 5% FOS (Meioligo W, Meiji Co., Tokyo, JAPAN) in drinking water during the same period. Six 12-week-old male mice that were fed a control diet (A02082003B, Research Diets Inc.) and purified water for 3 weeks and maintained under the same conditions served as the control group.

After the treatment period, 1.2 g of freshly collected feces were cryopreserved for analysis of the microbial flora, short-chain fatty acid and IgA concentration.

After the mice were sacrificed by cervical dislocation, alanine aminotransferase (ALT) levels were measured in serum samples prepared from venous blood. Mouse livers were extracted after perfusion with phosphate-buffered saline (PBS) containing 0.5% bovine serum albumin and 0.04% ethylenediaminetetraacetic acid (EDTA) (PBS buffer). Half of the liver was fixed in 4% paraformaldehyde (PFA) for hematoxylin–eosin (HE) staining and then evaluated pathologically according to the NAFLD activity score (NAS) [16]. The remainder of the liver was used for flow cytometric analysis. The extracted the ileum, colon, and half of the cecum were washed with PBS buffer and fixed in 4% PFA for HE and immunohistochemical staining. The rest of the cecum was used for flow cytometric analysis. The IgA density of feces was measured using a Mouse IgA ELISA Quantitation Kit (Bethyl Laboratories, Inc., Montgomery, TX, USA). This study was performed in strict accordance with the recommendations in the Guide for the Care and Use of Laboratory Animals of the National Institutes of Health. All efforts were made to minimize suffering. The protocol was approved by the Teikyo University’s School of Medicine’s Animal Ethics Committee (approval number: 14–030).

### Analysis of the microbial flora and short-chain fatty acids in feces

To identify bacterial species, the partial amino acid sequence of 16S rDNA was analyzed using the terminal restriction fragment length polymorphism method according to Nagashima’s method [17]. Briefly, 20 mg of feces were dissolved in 0.2 ml of distilled water and washed by centrifugation. The pellet was dissolved with 250 μl of TE buffer containing 100-mM Tris-HCl and 40-mM EDTA. After centrifugation with 0.6 g of DNA-extraction beads, the supernatant was collected and mixed with 150 μl of benzyl chloride and 50 μl of 10% sodium lauryl sulfate for 30 min at 50°C. After centrifugation with 150 μl of 3-M sodium acetate, the supernatant was collected and mixed with isopropyl alcohol for DNA extraction. Next, 0.5 U of HotStarTaq DNA polymerase (Qiagen, Tokyo, Japan) was added to 10 ng of DNA, and 16S rDNA was amplified by polymerase chain reaction using the 5′ terminal fluoro-labeled primers 516f and 1510r. After digestion with *BslI*, the fragment was analyzed using an ABI PRISM 3130xl DNA Sequencer (Applied Biosystems, Carlsbad, CA, USA) and GeneMapper (Applied Biosystems). The length of each fragment was distinguished using operational taxonomic units, and the peak area ratio was presented as a percentage. One gram of feces was pulverized under sterile conditions. Fecal short-chain fatty acids were analyzed using a Prominence high-performance liquid chromatography system (Shimazu Corp., Kyoto, Japan).

### Immunohistochemical staining

The 4% PFA-fixed tissue slices were subjected to an immuno-enzymatic method. Endogenous peroxidase activity was blocked using H_2_O_2_ containing Tris-buffered saline (TBS). Then, overnight incubation was performed with ×200 rabbit polyclonal anti-mouse zonula occludens-1 (ZO-1) (AVIVA Systems Biology Corp., San Diego, CA, USA), × 400 gout polyclonal anti-mouse IgA (Gene Tex Inc., Irvine, CA, USA), and × 200 rabbit polyclonal anti-mouse TNF-alpha (Lifespan Biosciences, Seattle, WA, USA). After washing with TBS-Tween, the tissue slices were incubated with a peroxidase-conjugated immune polymer for primal antibodies (Envison-PO for rabbit, Dako, Glostrup, Denmark) as a secondary antibody at room temperature for 1 h. After washing with TBS, 3,3′-diaminobenzidine was used for color development. Hematoxylin was used for nuclear counterstaining. In the ileum and colon, IgA-positive cells were counted in the range of 1 centimeter randomly selected. The mean number of IgA-positive cells was calculated. Ileal villus heights were measured in the same range, and mean heights were calculated.

### Flow cytometric analysis

Hepatic and cecal mononuclear cells were isolated as follows: Mouse livers and the cecum were filtered through a 40 μm cell strainer (BD Falcon, Durham, NC, USA) and re-suspended in PBS buffer. The solution was centrifuged at 500 rpm for 5 min, and the supernatant was centrifuged again at 1500 rpm for 5 min. After discarding the supernatant, the precipitate was dissolved in PBS buffer, layered over 1.077-g/ml Lymphoprep (Axis-Shield Proc. AS, Oslo, Norway), and centrifuged at 1500 rpm for 15 min. The mononuclear cell layer was collected and washed with PBS buffer. Viable cell counting was performed using trypan blue staining.

Cells (1 × 10^6^) were pre-incubated with 1 μl of purified anti-mouse CD16/32 (BioLegend, San Diego, CA, USA) in 24 μl of cell-staining buffer (BioLegend) at 4°C for 10 min and then incubated with 25 μl of a solution containing cell-staining buffer and appropriate quantities of fluorescein isothiocyanate-conjugated anti-mouse F4/80 and CD19 (BioLegend), phycoerythrin-conjugated anti-mouse CD14 (BioLegend), allophycocyanin (APC)/Alexa Fluor 750-conjugated anti-mouse CD11b (BioLegend), or APC-conjugated anti-mouse Toll-like receptor 4 (TLR4) and CD38 (BioLegend) at 4°C for 15 min in the dark. Rat IgG2a-kappa (BioLegend) was used as the isotype control. Stained cells were washed, suspended in 200 μl of PBS buffer, and dispensed into a 96-well round-bottom plate. Flow cytometric analyses were performed using a BD FACSArray flow cytometer (BD Immunocytometry Systems, San Jose, CA, USA) with FACSArray software (BD Immunocytometry Systems). Kupffer cells were identified as the F4/80^+^ CD11b^+^. B cells were identified as the CD19^+^ cell population. The mean fluorescence intensity (MFI) ratios of TLR4 and CD38 were calculated as the sample MFI divided by the isotype control MFI.

### Statistical analysis

Serum ALT levels, NAS, TLR4 MFI ratios, fecal short-chain fatty acid concentrations, numbers of IgA-positive cells in the small intestine, fecal IgA concentrations, and cecal length are presented as the mean ± standard error mean. Statistical analysis was performed by the nonparametric Mann–Whitney test and one-way ANOVA followed by Tukey post-test using GraphPad Prism version 6.0 for Macintosh (GraphPad Software, San Diego, CA, USA), and differences were considered significant at p < 0.05.

## Results

### MCD-induced dysbiosis and the effect of FOSs

**Fig 1:**
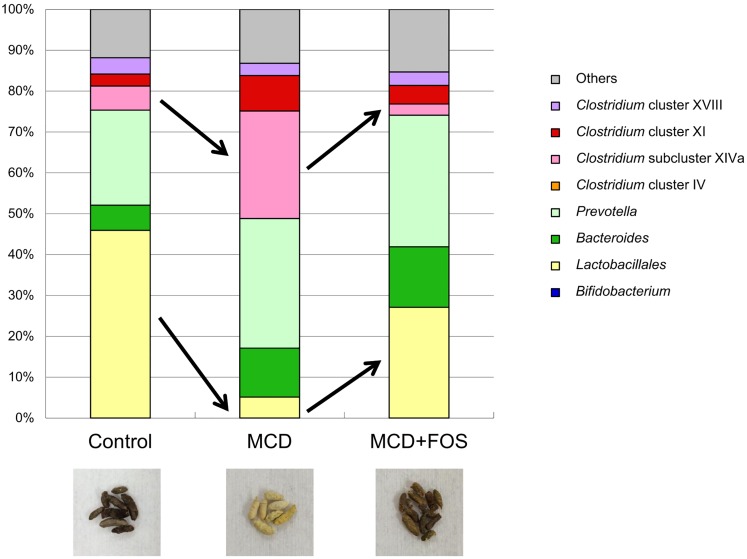
Terminal restriction fragment length polymorphism analysis of microbiological flora and macroscopic findings of feces from control, methionine–choline-deficient diet (MCD)-fed, and FOS-treated MCD-fed mice.

To confirm whether dysbiosis occurs because of MCD, we analyzed mice feces. Regarding the bacterial balance in feces of MCD mice compared with that in control mice’s feces, it was recognized that *Clostridium* cluster XI levels increased from 3% to 8.7%, and those of the *Clostridium* subcluster XIVa increased from 5.9% to 26.3%; on the contrary, counts of *Lactobacillales* spp. decreased from 45.9% to 5.1% (Fig 1). FOSs improved the bacterial levels, considering that after the treatment, the counts of *Clostridium* cluster XI and *Clostridium* subcluster XIVa decreased to 4.6% and 2.7%, respectively, and those of *Lactobacillales* spp. increased to 27.1%, in line with the bacterial balance of control mice. The color of feces in MCD mice was whitish, compared to brownish in MCD + FOS and control mice, reflecting a change of the bacterial balance.

### Histological findings of the liver

**Fig 2:**
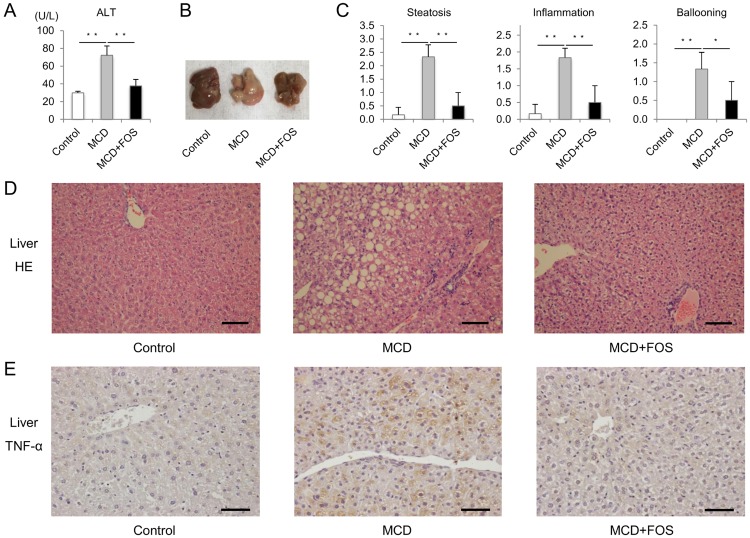
MCD-fed mice with or without FOS treatment. A. Mean values of serum alanine aminotransferase (ALT). *** p < 0.001. B. Macroscopic findings of the liver. C. Nonalcoholic fatty liver disease activity score. ** p < 0.01, * p = 0.03. Histological findings of the liver (D. hematoxylin–eosin stain, E. TNF-alpha stain. × 100. *Bar* = 100 μm).

To confirm whether MCD-induced NASH was improved by FOS, we analyzed serum ALT and liver tissue samples. Serum ALT levels (72.2 ± 10.6 U/l) were significantly higher in MCD mice than in control mice (29.8 ± 1.8 U/l, p = 0.0004; Fig 2a), and macroscopically, the liver was yellow (Fig 2b). Meanwhile, serum ALT levels (37.7 ± 7.3U/l) were significantly lower in MCD + FOS mice than in MCD mice (p = 0.0005), and the liver was brownish, as observed in control mice. Hepatic steatosis and inflammatory cell infiltrate were observed by HE staining of liver tissue in MCD mice (Fig 2d), whereas those changes were restrained in MCD + FOS mice. The NAS in MCD mice liver revealed scores of 2.3 ± 0.4, 1.8 ± 0.3, and 1.3 ± 0.4 points for steatosis, centrilobular hepatitis, and ballooning degeneration, respectively, whereas the value for each category was significantly decreased by 0.5 ± 0.5 points in MCD + FOS mice (steatosis, p = 0.003; centrilobular hepatitis, p = 0.005; ballooning degeneration, p = 0.03; Fig 2c). TNF-alpha staining of the liver of MCD mice showed positive staining of hepatocyte surrounding central vein; however, no staining was observed in FOS-treated mice (Fig 2e).

### Histological findings of the small intestine

**Fig 3:**
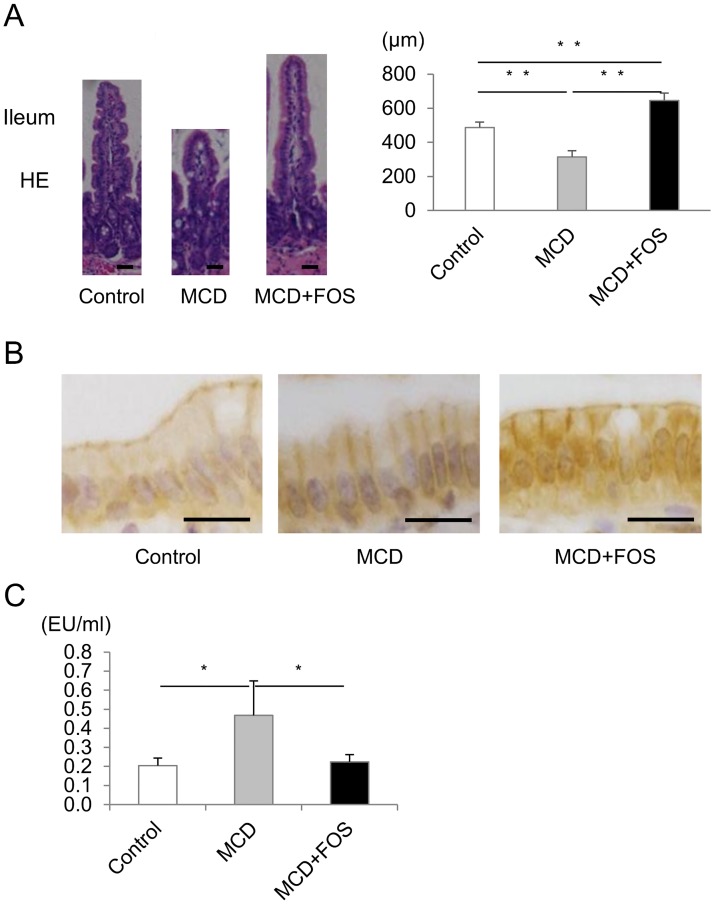
A. Mean villus heights (** p < 0.01) hematoxylin-eosin (HE, ×400. *Bar* = 50 μm) and B. zonula occludens-1 (ZO-1, ×600. *Bar* = 50 μm) staining in the ileal villus epithelium of methionine-choline-deficient diet-fed mice with or without the fructo-oligosaccharide treatment. C. Serumendotoxin level. * p < 0.05.

We hypothesized that MCD induces dysbiosis-mediated attenuation of intestinal barrier function, which would be improved by FOS; we analyzed villus heights and ZO-1 staining of the ileum. Remarkable changes were not observed in MCD mice via HE staining of the ileal tissues; however, villus extension was observed in MCD + FOS mice (Fig 3a). Villus heights were significantly higher in MCD + FOS mice (221.9 ± 8.6 μm) than in Control (192.1 ± 10.9 μm) and MCD mice (140.9 ± 25.2 μm) (p < 0.01). Length of the small intestine was longer in MCD + FOS mice (33.7 ± 5.6 mm) than in Control (30.1 ± 4.5 mm) and MCD mice (23.0 ± 2.7 mm) (p < 0.01). Length of the small intestine was also longer in MCD + FOS mice (7.0 ± 3.9 mm) than in MCD mice (5.1 ± 2.5 mm) (p = 0.02) but not significantly different from that in Control mice (7.3 ± 2.1 mm). When we examined the expression of tight junction proteins via ZO-1 staining, we recognized apical linear staining of the ileal epithelial cells and attenuation of the staining in MCD mice (Fig 3b). FOSs improved ZO-1 staining.

### Status of Kupffer cells

**Fig 4:**
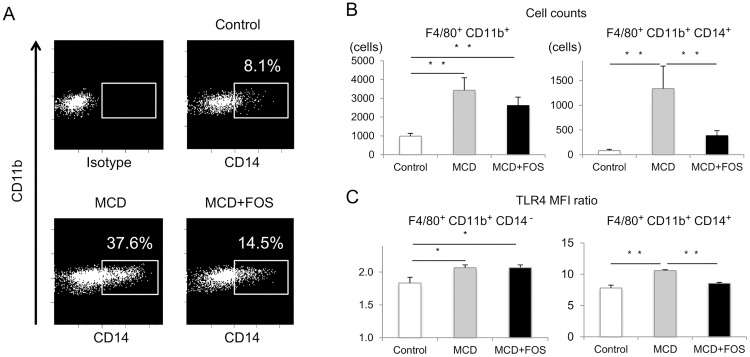
Flow cytometric analysis of F4/80^+^ CD11b^+^ Kupffer cells in the livers of MCD-fed mice with or without FOS treatment. A, Frequency of CD14^+^ Kupffer cells. B, Cell counts of total Kupffer cells and CD14^+^ Kupffer cells. * p < 0.05. C. Mean fluorescence intensity ratio of Toll-like receptor 4 in CD14^−^ and CD14^+^ Kupffer cells. * p < 0.05.

We hypothesized that MCD-mediated attenuation of intestinal barrier function could lead to higher LPS-induced Kupffer cell activation as these cells are activated by TLR4. Thus we measured total and CD14+ Kupffer cell numbers and MFI ratio of TLR4 using flow cytometry. The ratio of CD14^+^ cells among F4/80^+^ CD11b^+^ Kupffer cells was 8.1% ± 1.7% in control mice versus 37.6% ± 6.7% in MCD mice (p = 0.01) and 14.5% ± 2.8% in MCD + FOS mice (p = 0.01; Fig 4a). The number of total Kupffer cells was 984.7 ± 146.8 in control mice versus 3426.5 ± 663.3 in MCD mice (p = 0.003) and 2612.2 ± 452.5 in MCD + FOS mice (p = 0.07; Fig 4b). CD14^+^ Kupffer cell counts were 81.2 ± 27.2 in control mice versus 1335.7 ± 454.7 in MCD mice (p = 0.001) and 383.3 ± 101.4 in MCD + FOS mice (p = 0.002; Fig 4b). The MFI ratio of TLR4 in CD14^+^ Kupffer cells was 7.8% ± 0.5% in control mice, compared to 10.6% ± 0.1% in MCD mice (p = 0.04) and 8.5% ± 0.2% in MCD + FOS mice (p = 0.04; Fig 4c).

### Analysis of short-chain fatty acids in feces

**Fig 5:**
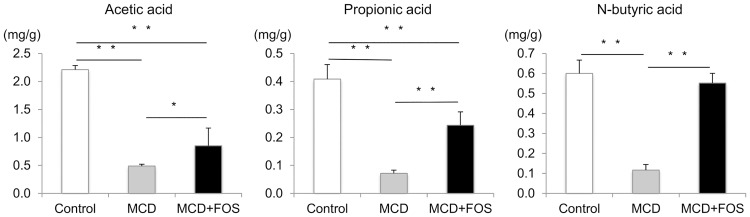
Fecal short-chain fatty acid concentrations of MCD-fed mice with or without FOS treatment. * p = 0.04, ** p < 0.01.

We hypothesized that MCD-mediated attenuation of intestinal barrier function was due to a reduction of *Lactobacillales*-produced short-chain fatty acids. Thus we measured short-chain fatty acids in feces. Upon analyzing short-chain fatty acids in feces, it was recognized that the concentrations of acetic acid (0.5 ± 0.1 mg/g, vs. Control’s 2.2 ± 0.1 mg/g; p = 0.003), propionic acid (0.1 ± 0.1 mg/g, vs. Control’s 0.4 ± 0.1 mg/g; p = 0.002), and n-butyric acid (0.1 ± 0.1 mg/g, vs. Control’s 0.6 ± 0.1 mg/g, p = 0.003) were significantly decreased in MCD mice (Fig 5). In MCD + FOS mice, the concentrations of acetic acid (0.9 ± 0.3 mg/g; p = 0.04) and propionic acid (0.2 ± 0.1 mg/g; p = 0.001) were significantly improved, whereas that of n-butyric acid (0.6 ± 0.1 mg/g; p = 0.002) was improved to control levels.

### Analysis of IgA-producing process

**Fig 6:**
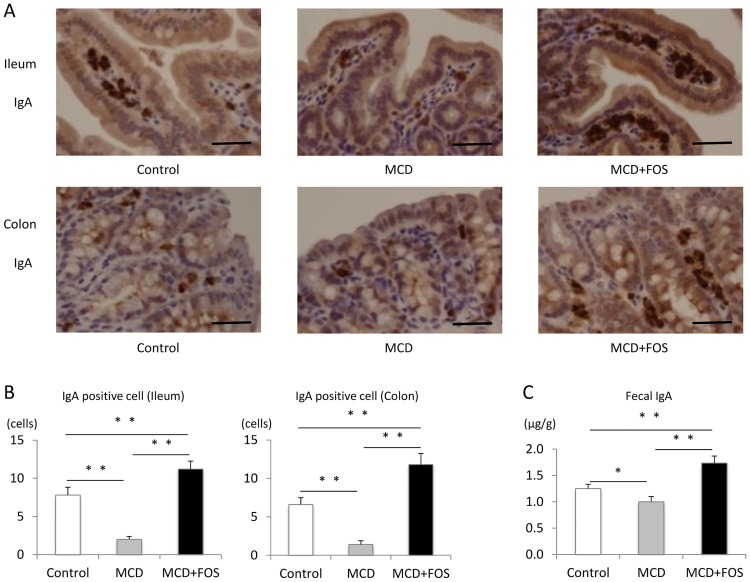
Immunohistochemical evaluation of IgA in ileal and colonic tissues and fecal IgA concentrations of MCD-fed mice with or without FOS treatment. A. Villus IgA staining in each group (× 600. *Bar* = 100 μm). B. Villus IgA-positive cells in each group. ** p < 0.01. C. Fecal IgA concentration in each group. * p = 0.01, ** p = 0.003.

We hypothesized that MCD-mediated attenuation of intestinal barrier function would include not only disruption of tight junctions but also that of mucosal immunity due reduced IgA production. Thus, we analyzed IgA-positive cells in ileal and colonic tissues. In the ileal tissue, there were more IgA-positive cells in MCD + FOS mice (155.2 ± 8.7 cells) than in Control (121.2 ± 10.9 cells) and MCD mice (98.4 ± 15.0 cells) (p = 0.001). It was similar in the colonic tissue: there were more IgA-positive cells in MCD + FOS mice (89.1 ± 13.3 cells) than in Control (79.3 ± 21.1) and MCD mice (66.4 ± 8.4 cells) (p = 0.03) (Fig 6b). It was recognized that the IgA concentration in feces was 1.3 ± 0.1 μg/g in control mice, compared to 1.0 ± 0.1 μg/g in MCD mice (p = 0.01; Fig 6c) and 1.7 ± 0.1 μg/g in MCD + FOS mice (p = 0.003).

**Fig 7:**
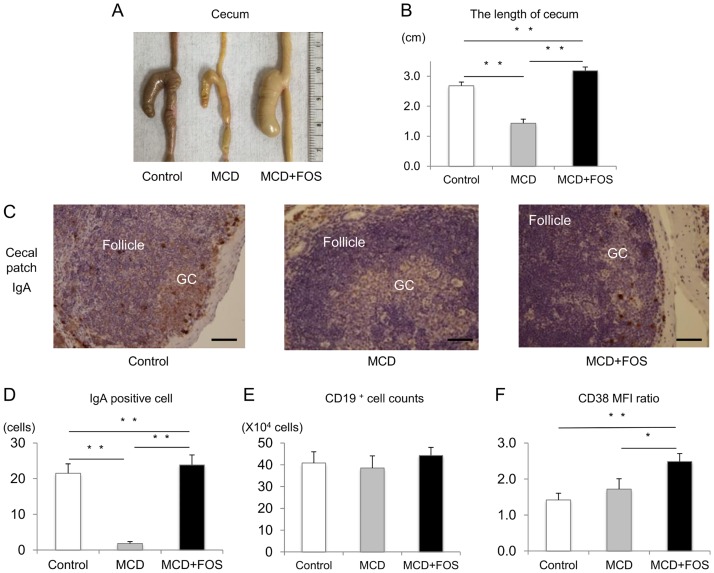
Cecal findings of MCD-fed mice with or without FOS treatment. A. and B. Macroscopic findings and mean length of the cecum in each group. ** p < 0.01. C. IgA staining of the cecal patch in each group (× 600. *Bar* = 100 μm). GC denotes germinal center. D. IgA-positive cell counts in each group. ** p < 0.01. E. Flow cytometric analysis of CD19^+^ B cell numbers (E) and mean fluorescence intensity ratio of CD38 (F) in each group. * p = 0.02.

MCD mice exhibited significant weight loss (18.8 ± 1.2 g, vs. Control’s 28.0 ± 1.3 g; p = 0.003) and shortening of cecal length (14.3 ± 0.1 mm, vs. Control’s 26.8 ± 0.1 mm; p = 0.004; Fig 7a and 7b). Although MCD + FOS mice had a similar weight as that of MCD mice (19.7 ± 1.1 g; p = 0.3), cecal length was longer in MCD + FOS mice than in MCD mice (31.8 ± 0.1 mm; p = 0.003). When we performed IgA staining of the cecal patch to investigate the IgA-producing cells, we observed IgA-positive cells in the follicle and germinal center of control and MCD + FOS mice, which we hardly observed in MCD mice (Fig 7c and 7d). To identify whether FOS can differentiate cecal patch B cells to IgA-producing cells, we analyzed B cells in cecal patch using flow cytometry. The number of CD19^+^ cells in the cecal patch showed no difference in each group (Fig 7e), but CD38, a B cell activation marker, was expressed more in MCD + FOS mice (2.5 ± 0.2) than in MCD mice (1.7 ± 0.3) (p = 0.02) (Fig 7f).

## Discussion

Mice that were fed an MCD developed steatohepatitis because large quantities of free fatty acids from white adipose tissue flow into the liver [18] and hepatic VLDL secretion is impaired [19]. As a limitation of this MCD-fed NASH animal model, the model does not reflect obesity or insulin resistance, but there is merit to causing NASH in the short term in comparison with high-fat diet feeding [20].

The existence of dysbiosis in NASH was reported as follows: decreased *Bacteroides* spp. and increased *Proteobacteria*, *Escherichia*, or *Clostridium coccoides* [21, 22] from an examination of patient feces and decreased *Lactobacillales* [23] in an MCD animal model.

In this study, we hypothesized that improvements of dysbiosis induced by FOSs can delay the onset of NASH, and examined this hypothesis in mice fed an MCD. Compared to control mice, *Clostridium* cluster counts were increased and those of *Lactobacillales* spp. were decreased in the feces of MCD mice. Concentrations of short-chain fatty acids and IgA in feces were also decreased. ZO-1 staining between ileal intraepithelial cells was diminished. In the liver, fibrosis was not observed because of the short duration of examination, but hepatic steatosis and inflammatory cell infiltration were observed. The percentage of CD14-positive Kupffer cells was increased, and TLR4 expression in these cells was upregulated.

**Fig 8:**
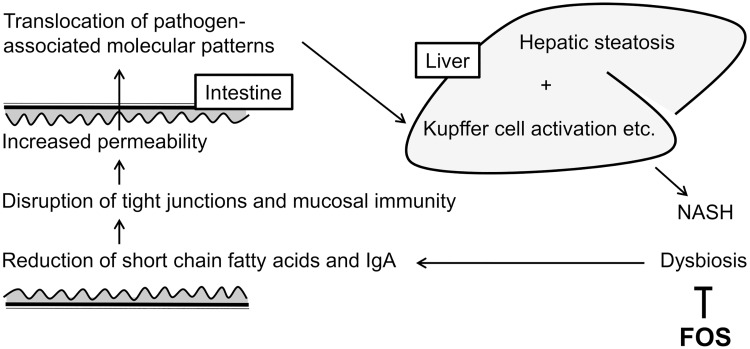
The supposed mechanism by which dysbiosis influences nonalcoholic steatohepatitis and its regulation by fructo-oligosaccharides.

As shown in Fig 8, the mechanism of intestinal barrier dysfunction is believed to be associated with decreased short-chain fatty acid production by intestinal bacteria, resulting in the depletion of energy sources for intestinal epithelial cells and a disordered homeostasis of intestinal mucosal immunity [7–9]. Through the disrupted intestinal barrier, it is assumed that PAMPs flow into the liver in large quantities [11, 12] and accelerate the production of inflammatory cytokines by Kupffer cells. Some reports identified high concentrations of endotoxin in the portal venous blood [11, 12] and enhanced expression of CD14 and TLR4 in Kupffer cells [13].

It is reported that FOSs increase *Bifidobacterium* and *Lactobacillales* spp. counts in the gastrointestinal tract. These bacteria produce short-chain fatty acids, which strengthen tight junctions by nourishing intestinal epithelial cells [9, 14, 24], as well as stimulate the differentiation of IgA-producing cells in the cecum and promote IgA secretion from intestinal mucosa [25, 26]. Our examination using MCD mice revealed that FOSs improved dysbiosis, increased *Lactobacillales* spp. counts, enhanced short-chain fatty acid production by intestinal bacteria, and improved ZO-1 staining in tight junctions. FOSs increased the number of IgA-positive cells in the germinal center of cecal patches and significantly reinforced IgA secretion by intestinal villi. Surprisingly, hepatic steatosis and inflammatory cell infiltration were decreased by FOS administration. The percentage of CD14-positive Kupffer cells and expression of TLR4 were also decreased. This report is the first to demonstrate that FOSs controlled the onset of NASH. This study was a small animal study with a short duration; in fact, no liver fibrosis was observed. This study is preliminary, and it should be followed by larger studies with longer durations to clarify the implications for human NASH. Together with the fact that we did not use another NASH model animal, these are limitations of our study; our findings support FOS administration as an effective treatment for NASH.

As a reason why steatohepatitis was improved by FOS administration, changes of the microbial flora may accelerate β-oxidation in the liver [27, 28]. In addition, short-chain fatty acids may both enhance intestinal barrier function and improve NASH directly. Adipose tissue expresses short-chain fatty acid receptors, which act to improve insulin resistance in the liver and muscle by inhibiting fat accumulation [29]. Furthermore, short-chain fatty acids act on the L cells of the intestinal tract, which promote GLP-1 secretion [30]. Further examination will reveal the direct action of FOSs in the liver.

Some methods to resolve dysbiosis have been reported. Recently, it was demonstrated that fecal microbiota transplantation (FMT) from healthy people is effective for treating patients with recurrent *Clostridium* infection [31]. The effectiveness of FMT in treating inflammatory bowel disease and irritable bowel syndrome, which are thought to involve dysbiosis, has also been suggested [32]. Serious side effects of FMT were not reported, but an evaluation of its effectiveness and safety is underway in Japan. Meanwhile, the replacement of useful bacteria as probiotics has been examined in NASH animal models. Velayudham *et al*. confirmed the downregulation of TLR4 and CD14 mRNA expression in the liver and the attenuation of hepatic fibrosis in MCD mice fed VSL#3, which is a probiotic that includes eight types of useful bacteria, for 10 weeks. However, they could not confirm a significant suppressive effect on hepatic steatosis and inflammation [33]. Endo *et al*. confirmed the reinforcement of tight junctions between intestinal epithelial cells and improvements of hepatic steatosis and fibrosis of choline-deficient/l-amino acid-defined diet-fed rats administered butyric acid-producing bacteria for 8–50 weeks [34]. However, it is difficult to reverse dysbiosis through the administration of single bacteria species.

To date, there is no approved therapy for improving NASH. Our study provided a potential dietary strategy for preventing and treating NASH. Prebiotics such as FOSs are present in onions, garlic, soybeans, and burdock, or are produced industrially as a syrup. They are more capable of being easily consumed habitually and continuously than probiotics. We believe FOSs can greatly contribute to a healthy life.

In conclusion, this study illustrated that in the MCD mouse model, dietary FOSs can restore the normal gastrointestinal microflora and normal intestinal epithelial barrier function and decrease steatohepatitis. The findings support the role of prebiotics such as FOSs in maintaining a normal gastrointestinal microbiome; they also support the need for further studies on the prevention or treatment of NASH using dietary FOSs.
